# Systemic Sequential Therapy of CisGem, Tislelizumab, and Lenvatinib for Advanced Intrahepatic Cholangiocarcinoma Conversion Therapy

**DOI:** 10.3389/fonc.2021.691380

**Published:** 2021-08-30

**Authors:** Yuan Ding, Xin Han, Zhongquan Sun, Jinlong Tang, Yingsheng Wu, Weilin Wang

**Affiliations:** ^1^Department of Hepatobiliary and Pancreatic Surgery, The Second Affiliated Hospital, Zhejiang University School of Medicine, Hangzhou, China; ^2^Key Laboratory of Precision Diagnosis and Treatment for Hepatobiliary and Pancreatic Tumor of Zhejiang Province , Hangzhou, China; ^3^Research Center of Diagnosis and Treatment Technology for Hepatocellular Carcinoma of Zhejiang Province, Hangzhou, China; ^4^Clinical Medicine Innovation Center of Precision Diagnosis and Treatment for Hepatobiliary and Pancreatic Disease of Zhejiang University, Hangzhou, China; ^5^Clinical Research Center of Hepatobiliary and Pancreatic Diseases of Zhejiang Province , Hangzhou, China; ^6^Zhejiang University Cancer Center, Hangzhou, China; ^7^Department of Pathology, The Second Affiliated Hospital, Zhejiang University School of Medicine, Hangzhou, China

**Keywords:** advanced intrahepatic cholangiocarcinoma, systemic sequential therapy, surgery, conversion therapy, next-generation sequencing

## Abstract

Intrahepatic cholangiocarcinoma (CCA), always diagnosed at an advanced stage in recent years, is of high aggression and poor prognosis. There is no standard treatment beyond first-line chemotherapy and no molecular-targeted agents or immune checkpoint inhibitors approved for advanced intrahepatic CCA. Hence, we firstly report an original therapeutic strategy for a 60-year-old patient diagnosed with intrahepatic CCA categorized as Stage IIIB (T3N1M0) by the American Joint Committee on Cancer staging system. After histopathological examination and next-generation sequencing, the patient was treated with four courses of novel systemic sequential therapy (intravenous gemcitabine 1,000 mg/m^2^ and cisplatin 25 mg/m^2^ on days 1 and 8; oral lenvatinib 8 mg/day from days 1 to 21; intravenous tislelizumab 200 mg on day 15). Then, the patient achieved partial response and was operated on right hemihepatectomy, cholecystectomy, and abdominal lymph node dissection. Without any perioperative complications, the patient was discharged from our hospital in perfect condition. Thereafter, the patient continued to use this new regimen 1 month after surgery for adjuvant therapy and was confirmed without recurrence when we followed up. In a word, we found an effective therapeutic regimen for preoperative advanced intrahepatic CCA conversion therapy, which may become a new approach in cancer treatment in the future.

## Introduction

Cholangiocarcinoma (CCA) is a heterogeneous group of cancers arising from the epithelial cells of intrahepatic and extrahepatic bile ducts (1). According to anatomic locations, intrahepatic cholangiocarcinoma (ICC) is one of the three CCAs, and the incidence of ICC has steadily risen in recent decades (2, 3). Radical resection (R0) that involves formal hepatectomy and portal lymphadenectomy is the best method among ICC patients for long-term survival (4). Unfortunately, because of highly aggressive malignancy, most of the patients are diagnosed at an advanced stage and even lose the chance to undergo surgery (2, 3, 5).

As more effective and novel chemotherapy, targeted therapies, and immunotherapy become available, multiple treatments can be chosen for the patients with advanced ICC (6). For instance, a double chemotherapy regimen using gemcitabine and cisplatin (CisGem) is supported by several recommendations (7, 8). Targeted agents, such as pemigatinib, show great efficacy in ICC therapy (9). In addition, on account of high genetic aberrations, most patients are sensitive to immunotherapy, taking pembrolizumab, for example (10). However, how to choose the most suitable therapy regimen is difficult, and most of them have not been approved at present.

Recently, whole-genome and transcriptome sequencing revealed the diversity of CCAs, offering a clearer understanding of carcinogenesis, classification, and treatment strategy (11). With the requirement of personalized therapies, multidrug combinations may also be the trend of novel treatments. Therefore, we describe a case that CisGem, lenvatinib, and tislelizumab were used to treat a patient for preoperative conversion therapy after genomic profiling in our hospital.

## Case Presentation

**Figure 1:**
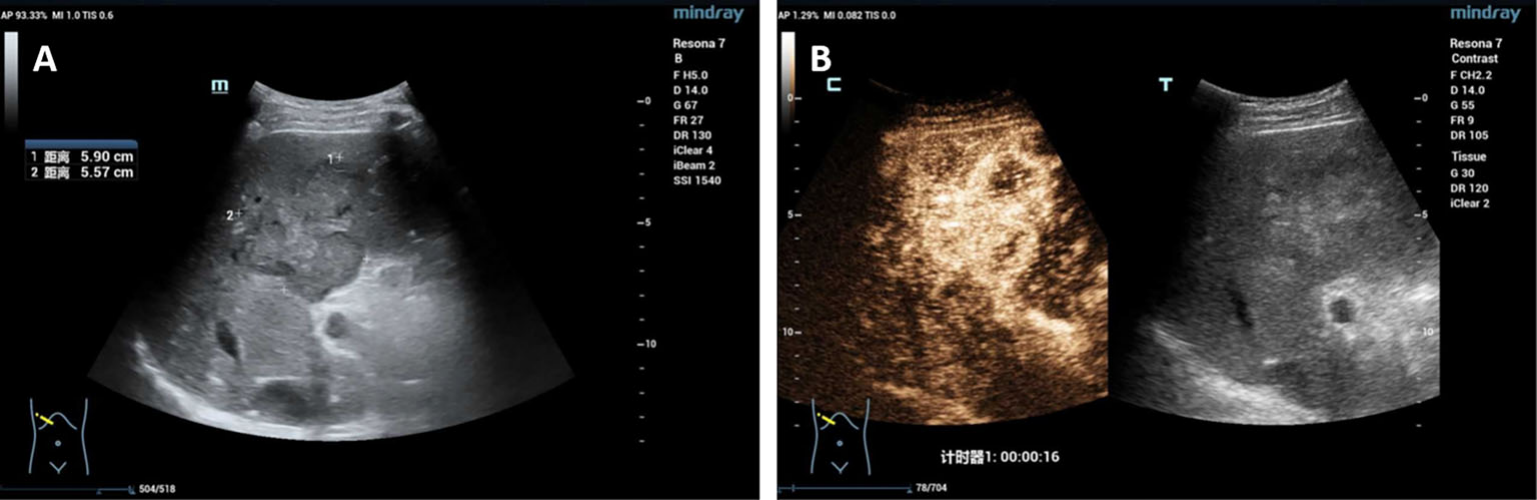
**(A)** Acoustic contrast of the viscera showed parenchymal hypoechoic masses; **(B)** the arterial phase showed uneven and high enhancement.

**Figure 2:**
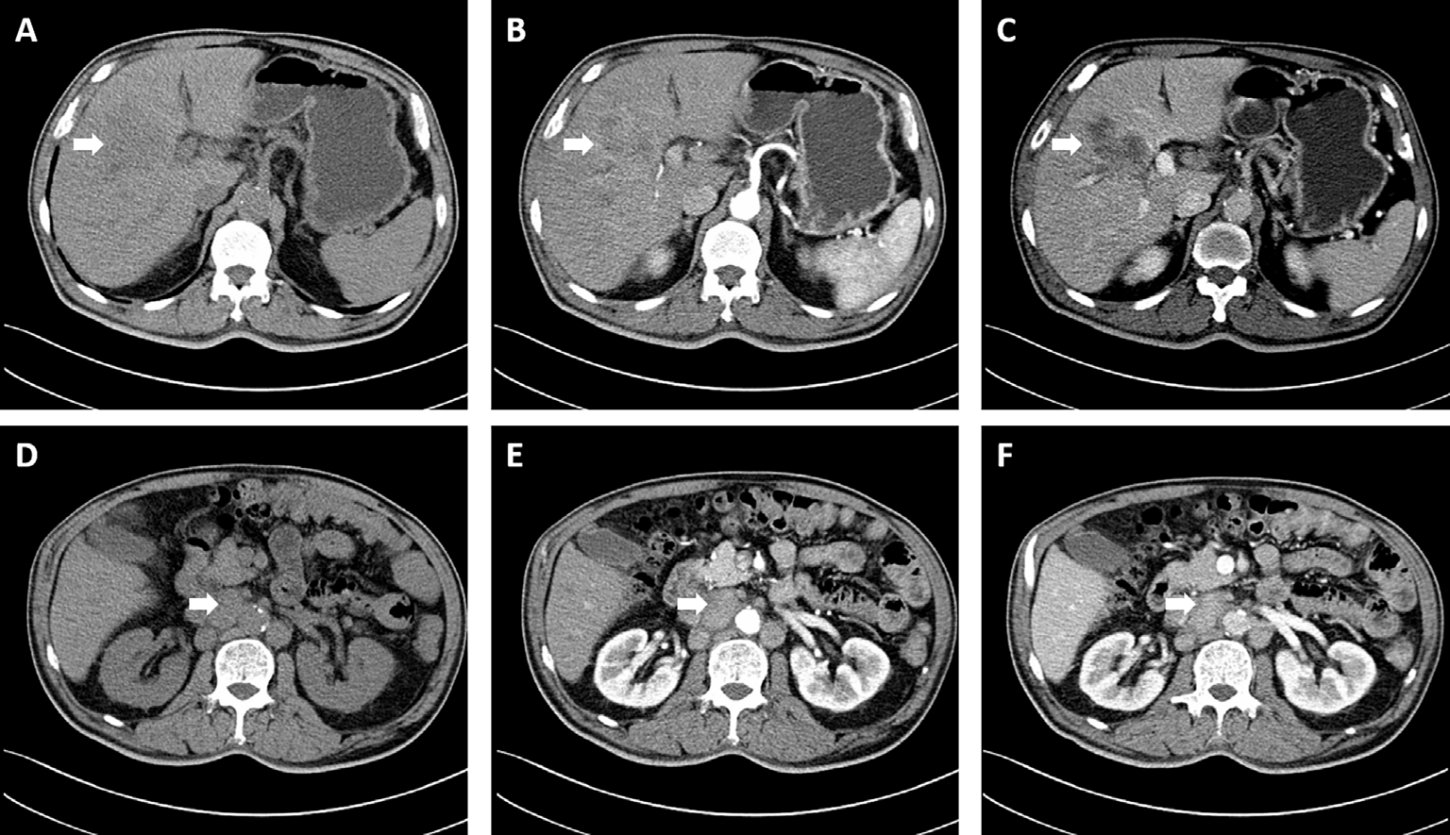
Computed tomography revealed an irregular morphology mass (49 mm * 39.6 mm) in the liver; the hepatic portal and retroperitoneal lymph nodes were enlarged. **(A, D)** Plain scan; **(B, E)** Arterial phase; **(C, F)** Portal venous phase. The white arrowheads direct the lesion or enlarged lymph nodes.

**Figure 3:**
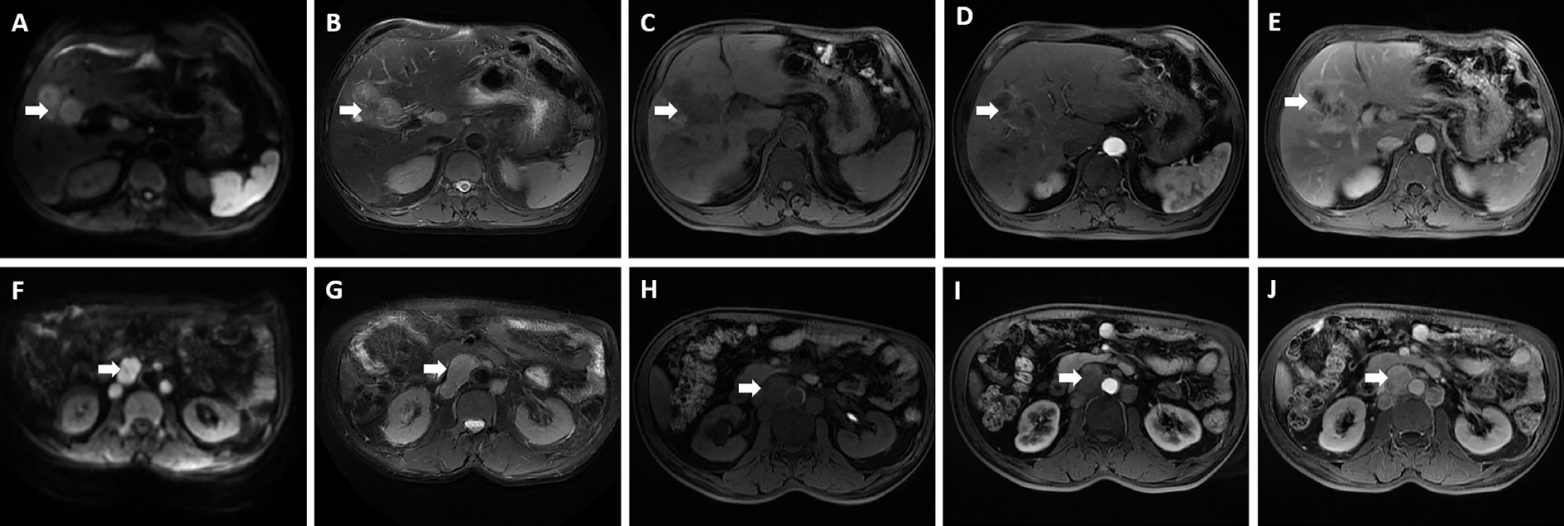
Magnetic resonance imaging showed an irregular morphology mass, with low signal in T1-weighted imaging, high signal in T2-weighted imaging, and limited diffusion in diffusion-weighted imaging (DWI); enlarged hepatic portal and retroperitoneal lymph nodes were also revealed—the larger one was 30.4 mm * 23.1 mm in size. **(A, F)** DWI; **(B, G)** T2-weighted imaging; **(C, H)** T1-weighted imaging; **(D, I)** The arterial phase; **(E, J)** The delayed phase. The white arrowheads direct the lesion or enlarged lymph nodes.

A 60-year-old male who was diagnosed with liver tumor by upper abdominal contrast-enhanced computed tomography (CT) in a local hospital presented to our department for further diagnosis and treatment on May 21, 2020, complaining of dull pain in the upper right abdomen without any symptoms of diarrhea, hematochezia, nausea, or vomiting for some weeks. Further inquiry revealed a history of hypertension, coronary heart disease, coronary stenting, and smoking, without any other history related to ICC such as primary sclerosing cholangitis and schistosomiasis. No other aberrations were noted, and physical examination was normal. Routine blood counts, coagulation function, and liver and renal function demonstrated normal levels except for alanine aminotransferase (ALT) (52 U/L) and aspartate aminotransferase (AST) (43 U/L). No clear elevation of tumor biomarkers including carcinoembryonic antigen (CEA), alpha fetoprotein (AFP), and carbohydrate antigen 125 (CA 125) was observed, while carbohydrate antigen 19-9 (CA 19-9) was a little higher (42.1 ng/ml). Hepatitis virus markers were all negative. Further examinations were performed after admission of the patient to our department. Acoustic contrast of the viscera showed parenchymal hypoechoic masses at liver segments IV and V, with blood flow signal by color Doppler flow imaging (CDFI) (**Figure 1**). Then, contrast-enhanced CT and magnetic resonance imaging (MRI) revealed an irregular-morphology mass (49 mm * 39.6 mm) in segments V–VIII of the liver; the hepatic portal and retroperitoneal lymph nodes were enlarged—the larger one (30.4 mm * 23.1 mm) lying between the inferior vena cava and the abdominal aorta (**Figures 2**, **3**). Based on the above information, the patient was diagnosed as having ICC clearly, which was classified as stage IIIB (T3N1M0) according to the American Joint Committee on Cancer (AJCC)/Union for International Cancer Control (UICC) staging system.

**Figure 4:**
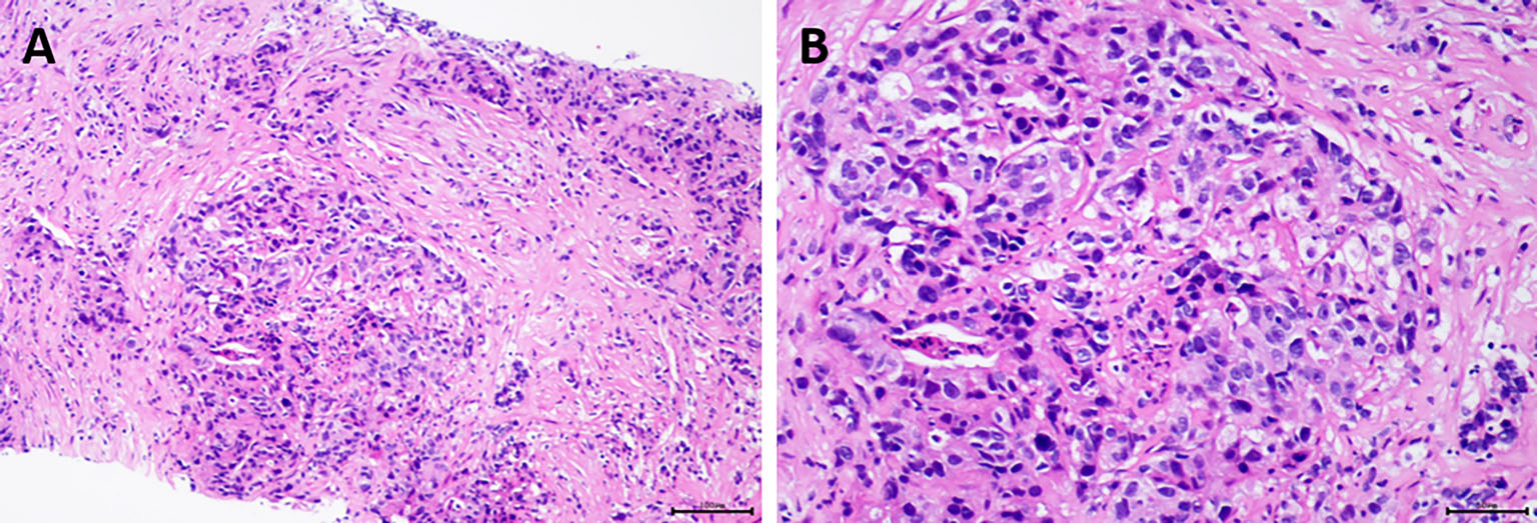
Pathological examination of the patient revealed poorly differentiated adenocarcinoma. **(A)** Hematoxylin and eosin (H&E), original magnification ×100; **(B)** H&E, original magnification ×200.

To further evaluate, we did liver biopsy and pathological examination. The results revealed a poorly differentiated adenocarcinoma (**Figure 4**). Immunohistochemical staining results were as follows: arginase-1 (-), Muc-1 (+), CK7 (+), CK20 (+), CDX2 (+), SATB2 (-), P53 (-), Ki-67 (60% +). In addition, next-generation sequencing (NGS) was performed at the same time; it showed the following: somatic mutation of gene: BRCA2 p.G2270, mutation frequency: 35.50%, and TP53 p.V73fs, mutation frequency: 50.00%. FGFR2 gene fusion was detected as well. The tumor mutational burden (TMB) was also determined to be 51.37 mut/Mb. Moreover, 48 unstable loci were revealed, and microsatellite instability (MSI) score was 0.5926. All of the above suggested that immunotherapy and targeted therapy might be effective against the patient’s tumor.

**Figure 5:**
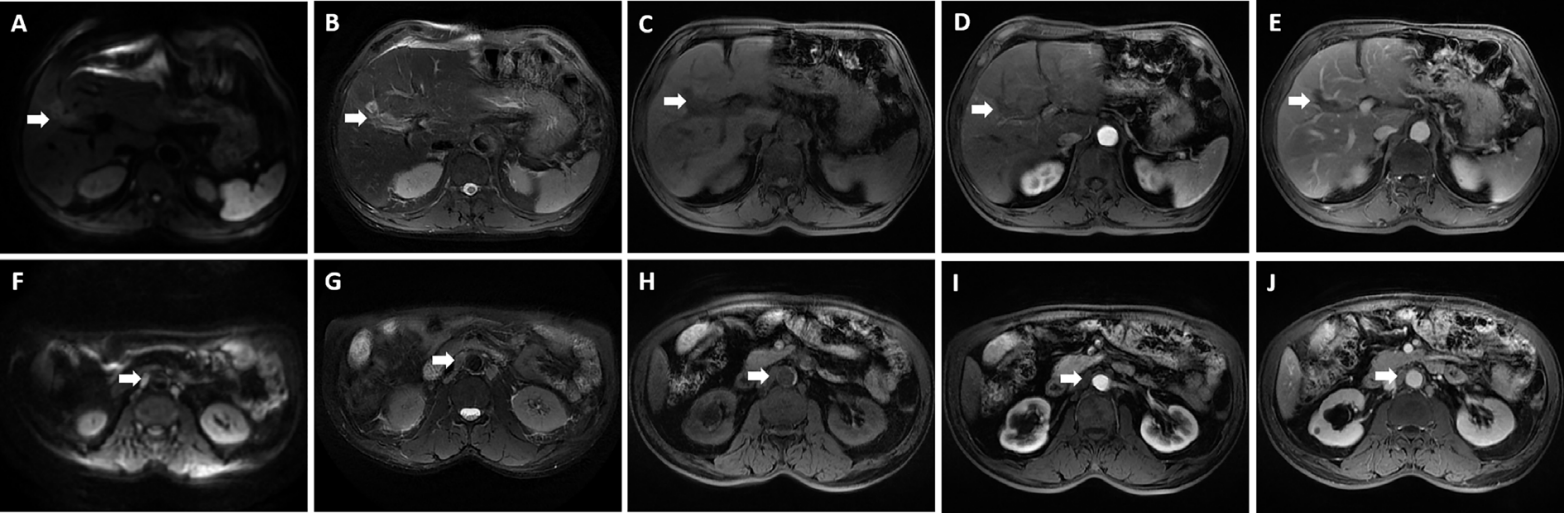
Preoperative magnetic resonance imaging was performed for the patient. The reduced lesion with clear boundary has low signal in T1-weighted imaging, high signal in T2-weighted imaging, and limited diffusion in diffusion-weighted imaging (DWI); enlarged hepatic portal and retroperitoneal lymph nodes were also revealed—the larger one was about 10 mm in diameter. **(A, F)** DWI; **(B, G)** T2-weighted imaging; **(C, H)** T1-weighted imaging; **(D, I)** The arterial phase; **(E, J)** The delayed phase. The white arrowheads direct the lesion or enlarged lymph nodes.

**Figure 6:**
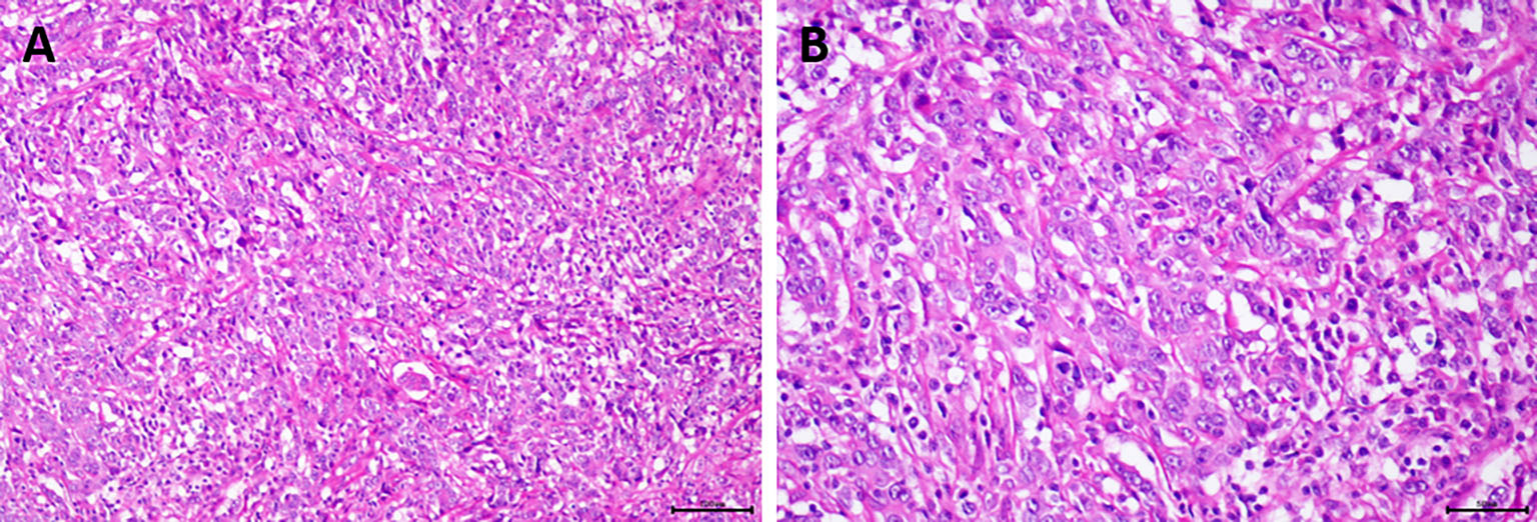
The postoperative pathological examination of the patient showed the tumor bed contained necrotic fibrous tissue proliferation, chronic inflammatory cell infiltration, cholesterol crystallization, and hemosiderin deposition, and low-grade intraepithelial neoplasia was seen in the surrounding bile duct. **(A)** Hematoxylin and eosin (H&E), original magnification ×100; **(B)** H&E, original magnification ×200.

**Figure 7:**
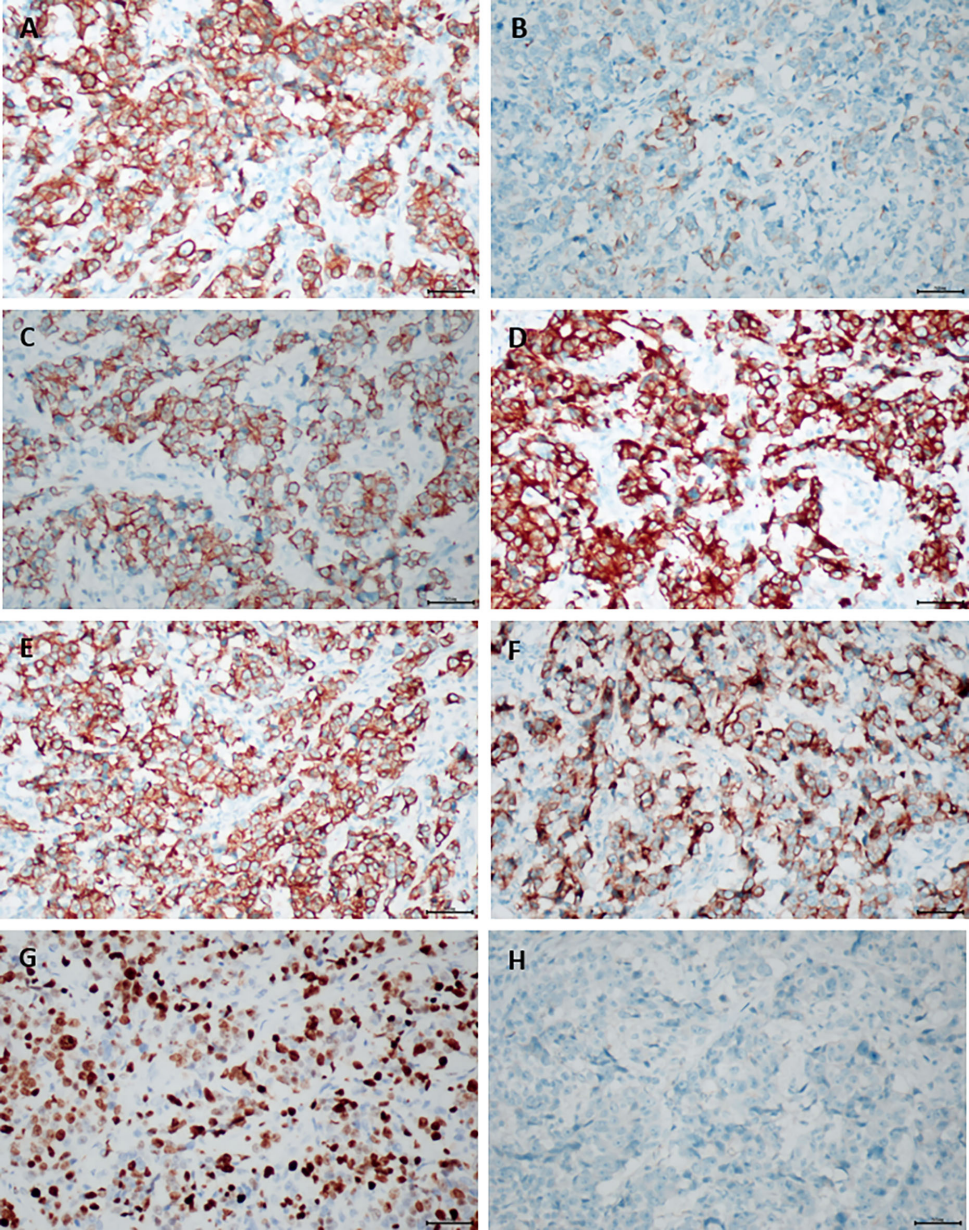
The postoperative immunohistochemical staining results, original magnification ×200. **(A)** CK (AE1/AE3); **(B)** CK7; **(C)** CK8; **(D)** CK18; **(E)** CK19; **(F)** MUc-1; **(G)** KI-67; **(H)** arginase-1.

Later, multidisciplinary treatment (MDT) was conducted to discuss the appropriate therapy. After MDT, a new therapeutic scheme, uniting CisGem, lenvatinib, and tislelizumab, was fully concerned. Hence, the patient was treated with four cycles of systemic sequential therapy (**Table 1**) without any obvious complications. Soon afterward, the measurement of target lesion was detected again by liver MRI on September 3, 2020. The lesion in the liver segments V–VIII was reduced, which was about 15 mm in diameter with clear boundary. Additionally, multiple lymph nodes metastasized in the hepatic portal and retroperitoneum, but they were obviously decreased compared with prior treatment (**Figure 5**). As expected, the patient achieved partial response (PR) successfully according to the standard RECIST 1.1 criteria. The CA 19-9 levels were reduced to normal range, in company with normal routine blood counts, coagulation function, other tumor markers, and liver and kidney function after one cycle of therapy. All these blood test results remained normal until four cycles of systemic sequential therapy finished. Subsequently, the patient underwent right hemihepatectomy, cholecystectomy, and abdominal lymph node dissection with enhanced recovery after surgery (ERAS) pathway in the Department of Hepatobiliary and Pancreatic Surgery. The postoperative pathological examination showed the tumor bed with necrotic fibrous tissue proliferation, chronic inflammatory cell infiltration, cholesterol crystallization, and hemosiderin deposition; was 4.5 cm * 2.5 cm in size, and low-grade intraepithelial neoplasia was seen in the surrounding bile duct; no clear tumor residue was found, and the Evans grade is IV; and the four hepatic portal lymph nodes dissected were negative, except one of Group 12 (**Figure 6**). Moreover, the postoperative immunohistochemical staining results suggested the following: CK (AE1/AE3) (+), CK7 (+), CK8 (+), CK18 (+), CK19 (+), MUc-1 (+), KI-67 index of 80%, arginase-1 (-) (**Figure 7**). Without any complications, the patient was discharged from the hospital in good condition.

**Table 1:** The systemic sequential therapy scheme.

Time (day)	Medication
Day 1	Intravenous gemcitabine 1,000 mg/m^2^ and cisplatin 25 mg/m^2^
Day 8	Intravenous gemcitabine 1,000 mg/m^2^ and cisplatin 25 mg/m^2^
Day 15	Intravenous tislelizumab 200 mg
Days 1–21	Oral lenvatinib 8 mg/day

**Figure 8:**
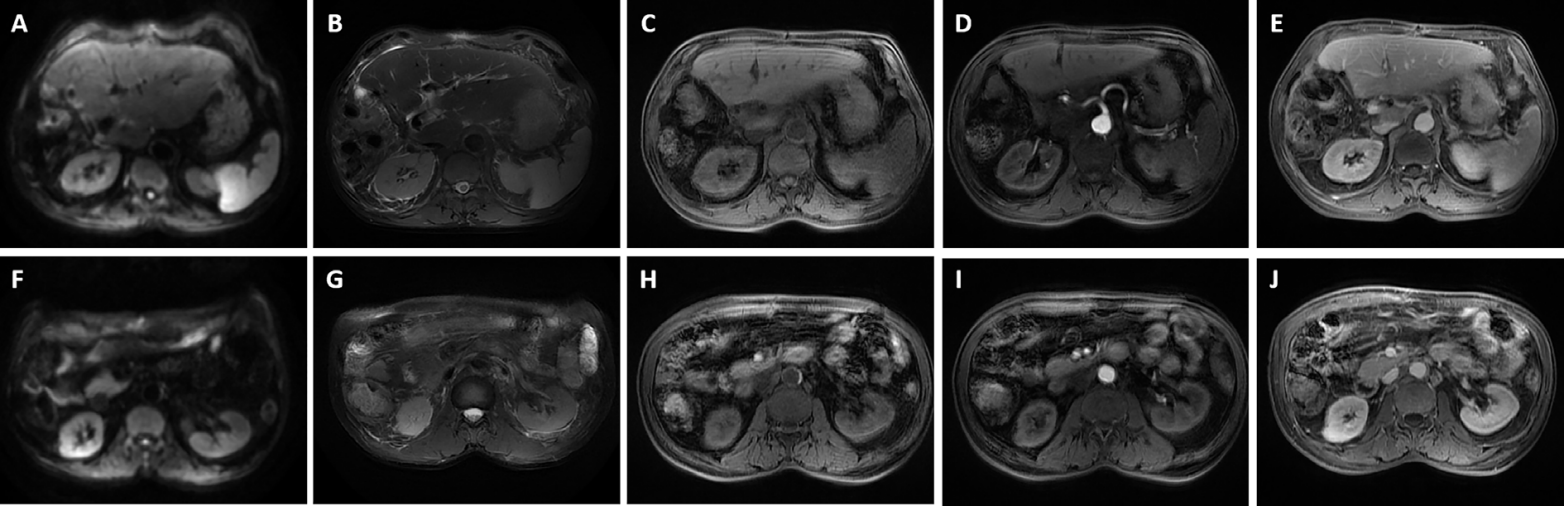
Magnetic resonance imaging was performed for the patient 1 month after surgery. It revealed postoperative changes of liver and gallbladder, a little exudation and effusion in the operative area. **(A, F)** Diffusion-weighted imaging (DWI); **(B, G)** T2-weighted imaging; **(C, H)** T1-weighted imaging; **(D, I)** The arterial phase; **(E, J)** The delayed phase.

**Figure 9:**
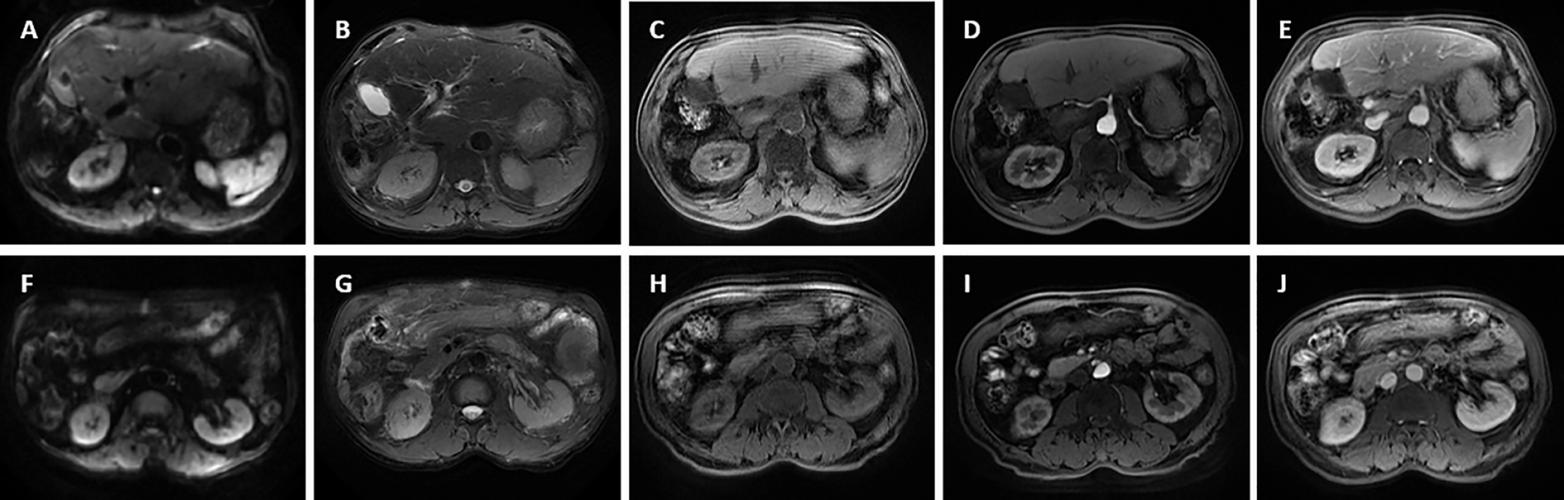
Magnetic resonance imaging was performed for the patient after four cycles of postoperative adjuvant chemotherapy. The tumor has disappeared without recurrence. **(A, F)** Diffusion-weighted imaging (DWI); **(B, G)** T2-weighted imaging; **(C, H)** T1-weighted imaging; **(D, I)** The arterial phase; **(E, J)** The delayed phase.

One month after surgery, MRI examination was performed again; it exhibited the following: postoperative changes of liver and gallbladder, a little exudation and effusion in the operative area (**Figure 8**). Furthermore, the patient used the new therapeutic regimen again for adjuvant therapy on October 24, 2020. The tumor has disappeared without recurrence after four cycles of adjuvant therapy, fortunately (**Figure 9**). Thereafter, the patient kept on using this original therapeutic regimen for further treatment. Up to now, the patient recovers very well without any severe side effects and recurrence during follow-up, which is more than 10 months after the operation.

## Discussion

It is the first time that we report a novel therapeutic regimen (uniting CisGem, lenvatinib, and tislelizumab) for preoperative advanced ICC conversion therapy according to pathological examination and NGS. The patient achieved PR after four cycles of treatment. Applying ERAS pathway in perioperation, the patient acquired R0 resection and recovered soon without any severe complications. Then, the patient continued to use the new therapeutic sequential regimen, and the patient recuperated well without recurrence and severe complications at postoperative follow-up.

ICC is relatively common to encounter in clinical practice, accounting for 12% of all primary hepatic carcinomas and approximately 25% of all CCAs (12, 13). Currently, only 30%–40% of patients have an opportunity to undergo surgery because of early metastasis; even after R0 resection, the postoperative recurrence rate in patients is approximately 60%–80% (14, 15). Nevertheless, the CisGem regimen (gemcitabine 1,000 mg/m^2^ and cisplatin 25 mg/m^2^ on days 1 and 8, every 21 days) is deemed to be the first-line treatment for advanced and metastatic ICC; the median overall survival (mOS) was 11.7 months and the median progression-free survival (mPFS) was 8.0 months (8). Unfortunately, as mOS is no longer than 1 year and no standard treatment beyond first-line chemotherapy is recommended now, more and more effective therapeutic strategies are to be explored.

In recent years, molecular-targeted therapy, immunotherapy, and multidrug combination therapy have shown interesting and attractive results (16, 17). Molecular-targeted agents, including vascular epidermal growth factor (VEGF) inhibitors, fibroblast growth factor (FGF) inhibitors, and isocitrate dehydrogenase (IDH) inhibitors, have provided new ideas for further treatment in advanced ICC (9, 17, 18). Lenvatinib, confirmed as an inhibitor of VEGF receptors 1–3, FGF receptors 1–4, platelet-derived growth factor (PDGF) receptor α, KIT, and RET, selectively inhibits receptor tyrosine kinases involved in tumor growth and angiogenesis (19, 20). It was successfully investigated in a multicenter phase III clinical trial, revealing that lenvatinib was non-inferior to sorafenib in overall survival in patients with untreated advanced hepatocellular carcinoma (HCC) along with great safety and tolerability (21). Up to now, the multikinase inhibitors sorafenib and lenvatinib are the only approved first-line treatments for advanced HCC by the US Food and Drug Administration (FDA) (22). Due to high expression of the immune checkpoint molecule programmed death-1 (PD-1) and its ligand (PD-L1), immunotherapy, a type of treatment regulating T-lymphocyte activity and enhancing the antitumor immune response, may effectively reduce tumor immune escape and become a promising adjuvant therapy for advanced ICC (23–25). For instance, the KEYNOTE-158 (NCT02628067) trial (n = 104) evaluated treatment with pembrolizumab monotherapy for advanced CCA patients and found that pembrolizumab was highly effective and safe; the mPFS, mOS, and overall response rate (ORR) was 2.0 months, 7.4 months, and 5.8%, respectively (10, 26). Tislelizumab, similar to pembrolizumab, is a PD-1 monoclonal IgG4 antibody of high affinity that is mainly used in hematological cancers and advanced solid tumors, which was conditionally approved after at least second-line chemotherapy in China (27, 28). Moreover, combination of immune checkpoint inhibitors and molecular-targeted agents has promising antitumor activity, taking lenvatinib plus pembrolizumab for example (29, 30).

Here, we highlight the usage of the first-line chemotherapy CisGem, the receptor tyrosine kinase inhibitor lenvatinib in combination with the anti-PD-1 drug tislelizumab for this advanced ICC patient after intense discussion in MDT. Firstly, tumor DNA mismatch repair (MMR) deficiency and high MSI are demonstrated with durability of responses to immune checkpoint inhibitors in some tumor types (31, 32). Similar to MMR and MSI, TMB is another emerging predictive biomarker for immunotherapy (33). In this tumor, deficiency in the MMR pathway was obviously detected, and replication errors with unstable abnormalities in short sequences of nucleotide were accumulated subsequently. Furthermore, the TMB was very high. Secondly, based on a phase II clinical study, lenvatinib was confirmed to have a tolerable safety profile for second-line treatment of CCA (34). Moreover, the cytotoxic cell death activity of chemotherapy would trigger antigen release, enhancing immune stimulation and improving the activity of PD-1/PD-L1-blocking agents (35). Therefore, we finally chose this systemic sequential therapeutic regimen for the ICC patient.

This case demonstrates that with a greater understanding of the molecular pathology and genomics of ICC, the therapy of tumors can gradually enter a more precise phase compared with other regimens. Furthermore, this report is a bold attempt and provides a glimmer of hope for advanced ICC patients, breaking through the bottleneck of traditional therapies. However, the long-term survival of this treatment has not been known. Whether the treatment can truly offer a clinical benefit and be approved needs further exploration with a large-scale randomized controlled trial in the future.

## Conclusion

We firstly reported an original systemic sequential therapeutic regimen for preoperative advanced ICC conversion therapy according to pathological examination and genomic profiling, providing an opportunity for radical resection. The patient recovered well with subsequent adjuvant therapy. Additional reliable studies with larger numbers of cases are needed to define certain efficacy and adverse effects for this disease.

## Author Contributions

(I) Conception and design: WW, YD and XH. (II) Administrative support: WW and YW. (III) Provision of study materials or patients: ZS and JT. (IV) Collection and assembly of data: XH, ZS and JT. (V) Data analysis and interpretation: YD and XH. (VI) Manuscript writing: All authors. All authors contributed to the article and approved the submitted version.

## Funding

This work was supported by the National Natural Science Foundation of China (No. 81773096, 82001673 and 82072650), Key Research and Development Program of Zhejiang Province (No. 2018C03085 and 2021C03121), and the Public Welfare Technology Research Project of Zhejiang Province (No.LGD19C040006).

## Conflict of Interest

The authors declare that the research was conducted in the absence of any commercial or financial relationships that could be construed as a potential conflict of interest.

The reviewer QX and the handling editor JC declared a shared affiliation, with no collaboration, with the authors, at the time of the review.

## Publisher’s Note

All claims expressed in this article are solely those of the authors and do not necessarily represent those of their affiliated organizations, or those of the publisher, the editors and the reviewers. Any product that may be evaluated in this article, or claim that may be made by its manufacturer, is not guaranteed or endorsed by the publisher.

## Abbreviations

CCA, cholangiocarcinoma; ICC, intrahepatic cholangiocarcinoma; HCC, hepatocellular carcinoma; R0, radical resection; CisGem, gemcitabine and cisplatin; CT, computed tomography; CEA, carcinoembryonic antigen; AFP, alpha fetoprotein; CA 125, carbohydrate antigen 125; CA 19-9, carbohydrate antigen 19-9; ALT, alanine aminotransferase; AST, aspartate aminotransferase; CDFI, color Doppler flow imaging; MRI, magnetic resonance imaging; AJCC, American Joint Committee on Cancer; UICC, Union for International Cancer Control; TMB, tumor mutational burden; MSI, microsatellite instability; MMR, mismatch repair; NGS, next-generation sequencing; MDT, multidisciplinary treatment; DWI, diffusion-weighted imaging; PR, partial response; ERAS, enhanced recovery after surgery; CR, complete response; mOS, median overall survival; mPFS, median progression-free survival; ORR, overall response rate; VEGF, vascular epidermal growth factor; FGF, fibroblast growth factor; IDH, isocitrate dehydrogenase; PDGF, platelet-derived growth factor; FDA, Food and Drug Administration; PD-1, programmed death-1.
